# Intrathecal Medication From Pain Pump Caused Prolonged Alteration in Mental Status Following Decompression of Severe Spinal Stenosis

**DOI:** 10.7759/cureus.24180

**Published:** 2022-04-16

**Authors:** Yamaan S Saadeh, Eleanor Smith, Juliana M Bilowus, Joseph R Linzey, Zoey Chopra, Paul Park

**Affiliations:** 1 Neurosurgery, University of Michigan, Ann Arbor, USA; 2 Neurosurgery, University of Michigan Medical School, Ann Arbor, USA

**Keywords:** intrathecal drug delivery, intrathecal pump therapy, pain, spinal stenosis, baclofen

## Abstract

Intrathecal drug delivery (IDD) has multiple indications, including chronic pain, spasticity, and spinal cord injury. Patients with an IDD device implanted who are undergoing decompressive spinal surgery may be at risk for intrathecal (IT) drug overdose in the perioperative setting. The present report describes a patient with an IDD device who underwent elective spinal surgery that was complicated by prolonged, severe alteration in mental status over several days, requiring discontinuation of his IT medications. The patient eventually returned to his neurological baseline by postoperative day 14. In the setting of severe spinal stenosis cranially in relation to an IDD device, consideration for weaning IT medications prior to elective surgery is recommended to avoid potential IT overdose. Patients undergoing weaning should be monitored for signs and symptoms of medication withdrawal.

## Introduction

Intrathecal drug delivery (IDD), first attempted in 1898 by German surgeon August Bier [1], is an important route of medication administration for chronic pain, spasticity, and spinal cord injuries [1,2]. Intrathecal (IT)-delivered medications have the advantage of bypassing the blood-brain barrier, which normally limits the transfer of many pain medications from the blood into the brain [3]. In these cases, oral medications must be given at high doses to achieve therapeutic dosing, which can result in adverse effects. An IT catheter generally delivers medication at a constant infusion via an implanted pump within the subcutaneous space [1,4]. IDD relies on drug delivery directly into the cerebrospinal fluid (CSF) surrounding the spinal cord, where the drug targets local opioid receptors in the dorsal horn and inhibits pain transmission. Morphine, ziconotide, and baclofen are approved by the FDA for IT use [1,5]. Off-label IT medications include bupivacaine, hydromorphone, fentanyl, and sufentanil [1].

IDD therapy is relatively safe. Most complications are related to surgical implantation or chronic opioid administration [2]. We present an unusual case of a patient who underwent elective spinal surgery that was complicated by prolonged, severe alteration in mental status over several days, likely because of severe spinal stenosis cranial to the location of the IT pain pump catheter preventing diffusion of IT medications within the spinal canal.

## Case presentation

**Figure 1:**
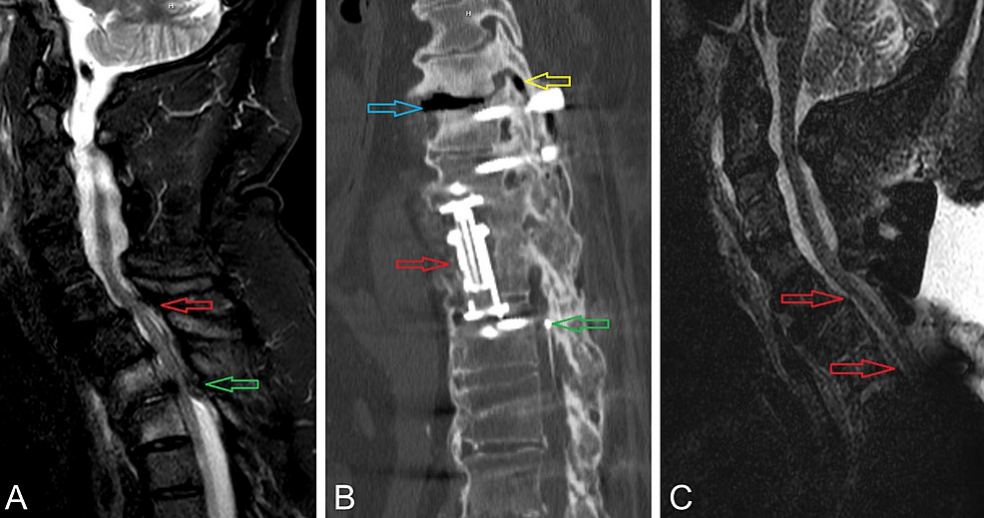
(A) Preoperative MRI T2 sagittal view demonstrates C7-T1 severe stenosis (red arrow) related to adjacent segment disease given the history of prior C3-C7 fusion. Severe central stenosis is also demonstrated at the T2-T3 level (green arrow). (B) Sagittal view of a CT study demonstrating prior T9-T10 corpectomy cage (red arrow) with T8-L2 fusion. The intrathecal catheter tip is visible, terminating within the spinal canal at the level of the T11 pedicle (green arrow). Air is also visible within the T7-T8 disc space (blue arrow) and the T7-T8 facet joint (yellow arrow), indicating the presence of significant mobility at that level. (C) Postoperative MRI T2 sagittal view demonstrating decompression of the C7-T1 and T2-T3 segments with the restoration of cerebrospinal fluid signal ventral to the spinal cord (red arrows).

This study was exempt from institutional review and patient consent was not required. A 66-year-old male presented to our neurosurgery clinic with a history of multiple spinal surgeries and placement of an IT pump for infusing ziconotide, baclofen, and hydromorphone. He reported two months of increasingly severe neck pain radiating to bilateral upper extremities with concomitant increases in dosages of his IT pain medications. On exam, he had full mobility and strength of his upper extremities and reduced function of his lower extremities. MRI revealed adjacent segment disease at C7-T1 and T7-T8, with additional high-grade stenosis at T2-T3 and T6-T7 resulting in multilevel high-grade spinal cord compression (Figure 1A). An IT catheter was visible within the spinal canal extending cranially to the T11 pedicle (Figure 1B). The patient underwent an extension of his previous fusion with C6-T8 screw fixation, along with C7-T3 and T6-T8 laminectomy, without intraoperative complication.

Postoperatively, the patient did not become sufficiently alert for extubation and was admitted to the ICU while intubated. On postoperative day (POD) one, the patient continued to be somnolent with depressed sensorium and did not arouse to noxious stimuli. His pupils were small and non-reactive. Head CT did not show a structural abnormality. EEG was negative for seizure and showed triphasic waves, which were thought to suggest a toxic/metabolic etiology. Naloxone was administered to presumptively treat for the possibility of persistent opioid effect, with no discernable response. On POD two, the patient remained intubated without the ability to follow commands, though he did begin to raise his arms in response to a sternal rub on examination. He developed hypotension requiring pressors.

Given his history of IDD, IT drug dosing was twice titrated down by 20%, without improvement. Given the persistent lack of improvement by POD two, the IT medicines were titrated down by 99%, and the patient was monitored closely for signs of baclofen withdrawal.

On POD three, the patient was able to open his eyes spontaneously. His pupils were round, 5 mm, and reactive bilaterally. He was able to localize pain in his upper extremities but remained in a state of depressed mental status and was highly agitated. By POD eight, the patient was awake and following commands. By POD 14, he returned to his neurologic baseline.

The patient’s IT baclofen was slowly titrated back up to the preoperative dose over the course of a few weeks. The patient was also supplemented with oral baclofen as needed to manage any symptoms of spasticity as his IT dose was being titrated. On POD four, the IT baclofen dose was raised from a minimal setting to 25% of the preoperative baseline. The dose was slowly titrated up to 50% of preoperative baseline at two weeks postoperatively, and back to full preoperative dose at one month postoperatively. IT ziconotide and hydromorphone were not resumed.

## Discussion

IDD is a well-established method for treating patients with chronic pain, spasticity, and spinal cord injuries [1,2,6,7]. Our patient had severe spinal stenosis proximal to the IT catheter causing a myelographic block on preoperative imaging. This myelographic block was suspected to also have caused decreased efficacy of his IT pain medication preoperatively. The potential impact of severe spinal stenosis located cranially to the level of an IT catheter has not been previously reported. The literature does describe differences in the distribution of IT medicine in the region immediately around the catheter, compared to more cranial regions [8-10].

In our patient, it is likely that the severe spinal stenosis causing a myelographic block also limited diffusion of IT medication in the cranial direction, necessitating higher IT medication doses for continued therapeutic effect over time. When these segments of severe spinal stenosis were decompressed, high doses of IT ziconotide, baclofen, and hydromorphone diffused suddenly without restriction toward the intracranial subarachnoid space. Although their concentration in the intracranial CSF would have been significantly less than in the region immediately adjacent to the catheter, the concentration was evidently sufficient to cause significant central nervous system depression via IT baclofen and opioid overdose.

There are innumerable reports within the literature describing the occurrence, sequelae, and management of IT baclofen overdose [11-14]. The symptoms of baclofen overdose have been reported to be significant neurological blunting, hypotension, fixed pupils, failure of spontaneous respiration, flaccid extremities, and unresponsive mental status [13,15]. With severe overdose, the response may model brain death [16]. Our patient demonstrated these signs, which at first were attributed to delayed recovery from anesthesia [15], though as the altered mental status persisted, other causes such as seizure, stroke, and hemorrhage were evaluated for and ruled out [17].

There are fewer reports describing ziconotide overdose within the literature, though the existing literature reports that IT ziconotide overdose events did not result in significant clinical sequelae requiring intervention [18]. A case series on six patients who experienced ziconotide overdose included a patient who unintentionally received 24 hours of dosing at 300 times the recommended dose. None of the patients in that study experienced any significant clinical sequelae [19].

No reports were found of an isolated IT hydromorphone overdose; however, there is a report of a patient who experienced a combined IT overdose with hydromorphone, clonidine, and bupivacaine causing hypertensive encephalopathy [20]. There are multiple case reports describing IT morphine overdose. One patient with a massive IT morphine overdose experienced severe hypertension, myoclonic seizure activity, and respiratory failure [21]. There are also reports of IT morphine overdose resulting in patient death [22].

Patients with IT medication therapy are at high risk for medication overdose [23]. The mainstay of therapy is recognition of the occurrence of an IT medication overdose, evaluation of the patient’s cardiopulmonary status and providing airway, breathing, and circulatory support as needed, and cessation of the IT medication therapy [24]. Depending on the specific medications involved, some potential reversal agents have been described, with mixed degrees of efficacy, and supportive therapy is the mainstay of treatment [25-27]. Cerebrospinal fluid removal and replacement via lumbar puncture and other approaches have also been described as a method for decreasing the concentration of overdosed medication within the spinal fluid [21,25,28].

To decrease the risk of IT medication overdose in patients with severe spinal stenosis and IDD catheter positioned caudal to the level of stenosis undergoing decompressive surgery, we propose the following preoperative measures. It is imperative to establish preoperatively the presence of IDD placement in patients with spinal stenosis via a thorough history, physical examination, and preoperative imaging. In the setting of severe spinal stenosis causing complete subarachnoid block radiographically, consideration for weaning IT medications prior to elective surgery is recommended to avoid potential IT overdose, particularly if there is a history of escalating dosage. Patients undergoing weaning should be monitored for signs and symptoms of medication withdrawal. IT baclofen withdrawal can be life-threatening and lead to refractory hyperthermia, rhabdomyolysis, renal failure, and disseminated intravascular coagulation, and more minor symptoms include itching, hypertension, hallucinations, and rebound spasticity [15]. Oral baclofen or intravenous Ativan may be administered in such cases where withdrawal seems likely [15]. Once the spinal stenosis has been decompressed, IT medications can be titrated back to the therapeutic range.

## Conclusions

Patients with an IDD device caudal to an area of severe spinal stenosis may benefit from weaning of IT medications prior to elective spinal decompression to prevent supratherapeutic doses of medication reaching the cranial subarachnoid space and causing significant alteration in mental status following surgery.
